# Alive Pathogenic and Saprophytic Leptospires Enter and Exit Human and Mouse Macrophages With No Intracellular Replication

**DOI:** 10.3389/fcimb.2022.936931

**Published:** 2022-07-11

**Authors:** Ignacio Santecchia, Delphine Bonhomme, Stylianos Papadopoulos, Pedro Escoll, Alexandre Giraud-Gatineau, Maryse Moya-Nilges, Frédérique Vernel-Pauillac, Ivo Gomperts Boneca, Catherine Werts

**Affiliations:** ^1^Institut Pasteur, Université Cité Paris, CNRS UMR6047, INSERM U1306, Unité de Biologie et Génétique de la Paroi Bactérienne, Paris, France; ^2^Institut Pasteur, Université Cité Paris, CNRS UMR6047, Unité Biologie des Bactéries Intracellulaires, Paris, France; ^3^Institut Pasteur, Université Cité Paris, CNRS UMR6047, Unité de Biologie des Spirochètes, Paris, France; ^4^Institut Pasteur, Université Cité Paris, Plateforme de Bio-imagerie Ultrastructurale, Paris, France

**Keywords:** Leptospira interrogans, Leptospira biflexa, macrophages, intracellularity, TLRs, high content confocal microscopy

## Abstract

*Leptospira interrogans* are pathogenic bacteria responsible for leptospirosis, a zoonosis impacting 1 million people *per* year worldwide. Leptospires can infect all vertebrates, but not all hosts develop similar symptoms. Human and cattle may suffer from mild to acute illnesses and are therefore considered as sensitive to leptospirosis. In contrast, mice and rats remain asymptomatic upon infection, although they get chronically colonized in their kidneys. Upon infection, leptospires are stealth pathogens that partially escape the recognition by the host innate immune system. Although leptospires are mainly extracellular bacteria, it was suggested that they could also replicate within macrophages. However, contradictory data in the current literature led us to reevaluate these findings. Using a gentamicin–protection assay coupled to high-content (HC) microscopy, we observed that leptospires were internalized *in vivo* upon peritoneal infection of C57BL/6J mice. Additionally, three different serotypes of pathogenic *L. interrogans* and the saprophytic *L. biflexa* actively infected both human (PMA differentiated) THP1 and mouse RAW264.7 macrophage cell lines. Next, we assessed the intracellular fate of leptospires using bioluminescent strains, and we observed a drastic reduction in the leptospiral intracellular load between 3 h and 6 h post-infection, suggesting that leptospires do not replicate within these cells. Surprisingly, the classical macrophage microbicidal mechanisms (phagocytosis, autophagy, TLR–mediated ROS, and RNS production) were not responsible for the observed decrease. Finally, we demonstrated that the reduction in the intracellular load was associated with an increase of the bacteria in the supernatant, suggesting that leptospires exit both human and murine macrophages. Overall, our study reevaluated the intracellular fate of leptospires and favors an active entrance followed by a rapid exit, suggesting that leptospires do not have an intracellular lifestyle in macrophages.

## Introduction

Leptospirosis is a worldwide neglected zoonosis caused by the pathogenic spirochetes *Leptospira interrogans.* In 5% to 20% of cases, leptospirosis can be fatal in humans and is responsible for 60,000 deaths annually (Costa et al., 2015). All vertebrates can be infected, and the symptoms are different in various hosts (Ellis, 2015). Among the animals that are naturally resistant to acute leptospirosis, mice and rats are asymptomatic carriers of the bacteria. Indeed, upon infection, the leptospires colonize the renal proximal tubules of mice and are then excreted in the urines (Ko et al., 2009; Ratet et al., 2014), contaminating soil and water and therefore contributing to the zoonotic cycle of the disease.

Upon infection, microbe-associated molecular patterns (MAMPs) of the bacteria are recognized by the innate immune system of the infected hosts. Receptors such as Nod receptors (NODs) and Toll-like receptors (TLRs) are implicated in the recognition of MAMPs and subsequent activation of antimicrobial responses. In the case of *Leptospira interrogans*, it was previously described that leptospires are classically not recognized by the innate immune system. Indeed, leptospires were shown to avoid TLR5 activation through the periplasmic localization of their endoflagella (Holzapfel et al., 2020). Also, leptospires escape NOD1/NOD2 by preventing the release of peptidoglycan fragments through its tight association with lipoprotein LipL21 (Ratet et al., 2017). Leptospires are among the few spirochetes to have a lipopolysaccharide (LPS) in their outer membrane, and leptospiral LPS is a virulence factor (Murray et al., 2010). In addition to the numerous leptospiral species described (Guglielmini et al., 2019; Vincent et al., 2019), hundreds of different serovars of leptospires have been characterized according to their different O antigens (Adler, 2015; Caimi and Ruybal, 2020; Bertasio et al., 2020). Nevertheless, the lipid A part of LPS is conserved among *L. interrogans* (Que-Gewirth et al., 2004; Eshghi et al., 2015; Novak et al., 2022). Leptospiral LPS possesses peculiar structural features (a methylated phosphate group and four amide bonds in the acyl chains) (Que-Gewirth et al., 2004), which most probably account for its escape from recognition by human TLR4 (Werts et al., 2001). However, the leptospiral LPS is recognized by mouse TLR4 (Nahori et al., 2005). Although it activates the classical MyD88 pathway (Bonhomme et al., 2020), we recently described that it escapes the endosomal TRIF pathway (Bonhomme et al., 2020). In addition, in both human and mouse cells, leptospires are potent agonists of TLR2, *via* their tri-acylated lipoproteins (Werts et al., 2001; Hsu et al., 2017). Recognition of leptospires by TLR4 and TLR2 was therefore shown to be essential to the resistance of the murine model to acute leptospirosis (Chassin et al., 2009).

In addition to their inflammatory functions, phagocytes such as macrophages are immune cells with potent microbicidal functions. First, they perform phagocytosis, during which pathogens adhere to the phagocyte surface through several receptors (CR3, FcR, Dectin 1) and get engulfed and degraded. Mechanistically, actin rewiring allows the formation of membranous pseudopod extensions around the cargo that are supported by lipid influx from the internal membrane network (Niedergang and Chavrier, 2004). Upon closure and maturation of the phagosome, it associates with lysosomal membrane proteins (LAMPs) and fuses with lysosomes, resulting in cargo degradation by enzymes such as cathepsins (Niedergang and Chavrier, 2004). In addition, macrophages can also degrade intracellular pathogens through xenophagy, a specific form of autophagy involving targeting of ubiquitinated cargo by adaptor molecules (Wang and Li, 2020). Such a mechanism can be triggered by NOD receptors and TLRs (Wang and Li, 2020), especially *via* TLR4-TRIF endosomal signaling upon LPS stimulation (Xu et al., 2007). Finally, macrophages are also equipped to kill both intracellular and extracellular microbes through the production of antimicrobial peptides (AMPs), reactive nitrogen species (RNS) such as nitric oxide (NO), and reactive oxygen species (ROS). Hydrogen peroxide (H_2_0_2_) and superoxide ${\text{O}}_{2}^{-}$ are among the most common forms of ROS and can be produced upon stimulation of the TLRs, including TLR2 and TLR4, together with the NADPH oxidase (NOX) enzymes (Ogier-Denis et al., 2008), to fight against pathogens.

Upon infection with *L. interrogans*, peritoneal macrophage depletion triggers the enhanced sensitivity of C57BL/6J mice to leptospirosis with slightly higher leptospiral loads (Tu et al., 1982; Isogai et al., 1986; Ferrer et al., 2018; Santecchia et al., 2019). Moreover, depletion of macrophages also results in higher chronic kidney colonization levels (Ferrer et al., 2018; Santecchia et al., 2019). However, the specific mechanisms by which macrophages recognize leptospires and contribute to their control remain poorly understood. Leptospires are mostly considered as extracellular bacteria, although the current literature has provided inconsistent evidence that non–opsonized leptospires could be found within macrophages (Merien et al., 1997; Liu et al., 2007; Li et al., 2007; Li et al., 2010; Toma et al., 2011; Zhao et al., 2013; Li et al., 2017). Interestingly, different studies showed that leptospires were either free in the cytosol or located within compartments, described as either endosomes, lysosomes, phagosomes, or generically “membranous” compartments (Merien et al., 1997; Liu et al., 2007; Li et al., 2007; Li et al., 2010; Toma et al., 2011; Zhao et al., 2013). Recently reviewed (Santecchia et al., 2020), these findings seem dependent on the pathogenicity of the leptospiral strains, on the cellular model (human *vs*. murine), and mostly on the infection protocol. Furthermore, the fate of internalized leptospires has also been addressed in several studies but remains controversial. A study concludes that leptospires replicate efficiently in the cytosol of human macrophages only, whereas murine macrophages can kill them (Li et al., 2010). Furthermore, the killing mechanism is attributed to the production of ROS in murine cells (Li et al., 2017). However, another publication conversely showed phagocytosed leptospires in both human and murine cells (Zhao et al., 2013). The intracellularity of the leptospires and their fate in human and murine macrophages therefore remain open questions.

We first used a gentamicin-protection assay to unequivocally assess the internalization of leptospires in macrophages *in vivo* and *in vitro*, using both transmission electron microscopy and high-content confocal microscopy. Then, we further investigated the intracellular fate of bioluminescent pathogenic *Leptospira interrogans* and saprophytic *Leptospira biflexa* in both human and murine macrophages. Finally, we addressed the role of the main classical microbicidal mechanisms of macrophages against leptospires, namely, phagocytosis, autophagy, and ROS and RNS production.

## Materials and Methods

### *Leptospira interrogans* Cultures

All *Leptospira interrogans* used in this work [serovar Manilae strain L495 and bioluminescent derivative MFLum1 (Ratet et al., 2014), serovar Copenhageni strain Fiocruz L1-130, serovar Icterohaemorrhagiae strain Verdun virulent Clone 3 (Vernel-Pauillac et al., 2021)] and *Leptospira biflexa* (serovar Patoc strain Patoc I and bioluminescent derivative PFLum7 (Ratet et al., 2014)) were grown in Ellinghausen–McCullough–Johnson–Harris (EMJH) medium at 28°C without agitation and diluted weekly, or twice a week for *L. biflexa*, to obtain exponential-phase growth culture at the time of experiments. Virulence of strains was controlled *in vivo* in mice according to a clinical chart (Vernel-Pauillac et al., 2021) including, when possible, measures of bioluminescence at day 3 post-infection. For infection, leptospire cultures were centrifuged (3,250 g, 25 min), resuspended in endotoxin-free PBS (Lonza), and enumerated using a Petroff–Hauser chamber and bacterial concentration was adjusted accordingly. To inactivate bacteria [hereafter called “heat-killed” (HK)], leptospires were heated at 56°C for 30 min.

For fluorescent labeling, 10 ml of exponential culture was centrifuged (3,250 g, 25 min) and the pellet was resuspended in the same volume of PBS (Lonza) supplemented with 10 µM CFSE (Sigma). The bacterial suspension was incubated at room temperature for 30 min protected from light and washed one time with 20 ml of PBS before infection.

Alamar Blue was used for the evaluation of leptospiral viability. Bacteria were plated at 1 × 10^6^ leptospires/well in 96-well plates in 180 µl of EMJH medium (Mouville and Benaroudj, 2020). Next, 20 µl of Alamar Blue (Thermo Fisher Scientific, Waltham, MA, USA) were added to the cultures, and the plates were incubated for 72 h at 30°C.

### *In Vivo* Internalization Experiment

Adult female C57BL/6J mice were injected *via* intra-peritoneal route with 5 × 10^7^ CFSE-stained *L. interrogans* serovar Manilae strain L495. Next, 1 h post-infection mice were euthanized, and peritoneal content was recovered as previously described (Santecchia et al., 2019). Briefly, the skin was peeled and clamped, followed by the injection of 2 ml of RMPI with 10% v/v heat-inactivated fetal calf serum (HI-FCS, Gibco, Grand Island, NY, USA) and 100 µg/ml gentamicin (Gibco). Next, an incision was made on the pre-clamped skin to recover the peritoneal content. Peritoneal cells were distributed into optical-grade transparent-bottom black 96-well plates in the same medium and left to adhere for 90 min. Next, the plates were washed two times, followed by a 10-min fixation in 4% w/v *para*formaldehyde (pFA). After fixation, the cells were blocked with 5% w/v BSA, 0.06% w/v saponin, and 2.5 µg/ml FcBlock (eBioscience, San Diego, CA, USA) during 1 h, followed by staining with 2.5 µg/ml APC anti-mouse F4/80 (clone BM8, BioLegend, San Diego, CA, USA) or mouse IgG2b isotype control APC-coupled (BioLegend) during 1 h. Staining was followed by three washes in 1% w/v BSA, counterstained with 1 µg/ml DAPI for 10 min, washed two times, and imaged using high-content microscopy as described elsewhere.

### Ethics Statements

All protocols were undertaken in compliance with EU Directive 2010/63 EU and the French regulation on the protection of laboratory animals issued on February 1, 2013. They are part of project number # 2014-0049, which was approved by the Institut Pasteur ethics committee for animal experimentation (Comité d’Ethique en Expérimentation Animale CETEA registered under #89) and was authorized under #8562 by the French Ministry of Research, the French Competent Authority.

### Cell Culture

The RAW264.7 murine macrophage-like cell line was cultured in antibiotic/antimycotic-free complete RPMI containing 1 mM L-glutamine (Lonza, Walkersville, MD, USA) and supplemented with 10% v/v heat-inactivated fetal calf serum (HI-FCS, Gibco), 1 mM non-essential amino acids (MEM, Gibco), and 1 mM sodium pyruvate (NaPy, Gibco). The cells were seeded in 96-, 24-, and 12-well plates (TPP) in 200 µl, 1 ml, or 2 ml, respectively.

Bone marrow-derived macrophages (BMDMs) were obtained from femurs of C57BL/6J mice: WT (Janvier, Le Genest, France), TLR4 ko, TLR2 ko, TLR2/4 dko, and MyD88^-/-^/TRIF*^LPS2^* (MyD88/TRIF dko) [bred by the Institut Pasteur animal facility and previously described (Chassin et al., 2009)]. After euthanasia by cervical dislocation, the head of each bone was removed, and 2 ml of medium was passed through the bones using a 22-G needle to flush out the bone marrow. Bone marrow cells were centrifuged (300 g, 7 min) and treated with red blood cell lysis buffer (Sigma-Aldrich, St. Louis, MO, USA) for 10 min, followed by another centrifugation. The cells were then resuspended in complete RPMI, enumerated, and frozen at -80°C in 10% v/v DMSO in the same medium. Unfrozen cells (5 × 10^6^) were seeded in 100-cm^2^ cell culture dishes in 12 ml of complete RPMI supplemented with 10% v/v L929 cell supernatant and 1× PenStrep (Gibco). Cells were incubated at 37°C, 5% CO_2_. At day 3, 3 ml of the same medium was added. At day 7, the cells were collected by scraping in cell dissociation buffer (Gibco), centrifuged, enumerated, and seeded in antibiotic/antimycotic-free medium in 96-, 24-, and 12-well plates (TPP) in 200 µl, 1 ml, or 2 ml, respectively.

The THP-1 monocyte cell line was cultured in antibiotic/antimycotic-free complete RPMI, and the cells were differentiated for 48 h with 20 ng/ml of phorbol 12-myristate 13-acetate (PMA, InvivoGen, San Diego, CA, USA) in 96-, 24-, and 12-well plates (TPP) in 200 µl, 1 ml, or 2 ml, respectively. Cells were then washed three times with PBS and allowed resting for 24 h before infection.

Whenever indicated, cells were pretreated with inhibitors for 30 min with 25–50 µM (2R,4R)-4-aminopyrrolidine-2,4-dicarboxylic acid (APDC, Enzo, Farmingdale, NY, USA), 10 µM cytochalasin D (CytoD, Sigma-Aldrich), 10 nM bafilomycin A1 (BafA1, Sigma-Aldrich), or 10 µM S-methylisothiourea sulfate (SMT, Sigma-Aldrich). For lipopolysaccharide stimulation, cells were treated with *Escherichia coli* LPS (EB, InvivoGen) at 1 µg/ml during the assay.

### Gentamicin-Protection Assay

RAW264.7 cells or BMDMs were plated the day before infection at 0.3 × 10^6^ or 1 × 10^6^ cells/ml, respectively, and were incubated overnight at 37°C, 5% CO_2_. For infection, 50% of the media was removed and replaced by bacterial suspension and/or corresponding drug treatment in complete RPMI medium. After 1 h of infection, the cells were washed three times with media, and complete RPMI supplemented with 200 µg/ml gentamicin (Gibco) was added to the cells for 2 h. After antibiotic treatment, the cells were washed three times and cultured in antibiotic-free RPMI, until the indicated time points where intracellular bacteria were recovered by lysing macrophages or the cells fixed for fluorescence microscopy and cytometry as indicated elsewhere. To recover intracellular bacteria, the cell supernatant was removed, and to lyse the cells, 350 µl of sterile distilled water (Gibco) was added for 15 min at room temperature with mild agitation. Cell lysates were then flushed and inoculated in tubes containing 5 ml of pre-warmed (30°C) EMJH media. Bacterial load was immediately determined by measuring bioluminescence in the cultures or indirectly assessed by monitoring leptospiral growth by optical density measurements at 420 nm. Bioluminescence of MFlum1 and PFlum7 *L. interrogans* was measured on a Centro X100 luminometer (Berthold, Bad Wildbad, Germany) after addition of XenoLight D-luciferin (PerkinElmer, Waltham, MA, USA), as described (Ratet et al., 2014).

### High-Content Immunofluorescence Microscopy Analysis

RAW264.7 cells were plated the day before infection at 0.1 × 10^6^ cells/ml and were incubated overnight at 37°C, 5% CO_2_. For infection, 50% of the media was removed and replaced by bacterial suspension. After infection with CFSE-labeled leptospires and gentamicin assay, the internalization of the leptospires was assessed by high-content microscopy. The cells were fixed in 4% w/v pFA for 10 min, followed by three washes with PBS. Cells were blocked for 1 h in PBS with 2.5% w/v bovine serum albumin (BSA) and 2.5 µg/ml FcBlock (eBioscience). Cells were then stained for 30 min in PBS 1% w/v BSA and 0.05% w/v saponin with 5 UI/ml of phalloidin-AlexaFluor-647 and 1 µg/ml of DAPI, staining actin, and nuclei, respectively. The cells were washed three times with PBS and finally kept in PBS for imaging using Opera Phenix HCS (Perkin Elmer) using confocal settings with a ×63 water-immersion objective. Light source was kept at 20% to 50% power, and exposure time was set for each experiment to obtain intensities around 1,000–5,000. Automated acquisition was launched to acquire a minimum of 500–1,000 cells/well in technical triplicates for each experiment. Automated image analysis workflow and puncta quantification was performed using Columbus software (PerkinElmer), with the following script: i) import data; ii) find nuclei with the DAPI signal; iii) find cytoplasm with either F4/80 or phalloidin staining; iv) find spots using method C; v) formulate classical outputs: number of spots/cells; vi) formulate calculated outputs: % spot positive cells; vii) save script and run batch analysis; and viii) export data.

### Electron Microscopy

BMDMs were plated the day before infection at 1 × 10^6^ cells/ml and were incubated overnight at 37°C, 5% CO_2_. For infection, 50% of the media was removed and replaced by bacterial suspension. After infection with leptospires, BMDMs were washed and fixed after a 1 h infection with *L. interrogans* serovars Manilae strain MFLum1 in 2.5% w/v glutaraldehyde (Sigma) in 0.1 M PHEM buffer (120 mM PIPES; 50 mM HEPES; 20 mM EGTA; 4 mM MgCl_2_) at room temperature for 1 h and then overnight at 4°C. Cells were then washed three times in PHEM buffer before quenching with 50 mM NH_4_Cl in PHEM buffer. Cells were then scrapped and centrifuged before resuspension in 20 µl of agar LMP. Post–fixation was performed with 1% w/v osmium tetroxide (Merck) in PHEM buffer during 1 h at room temperature. Dehydration was gradually performed with 25%, 50%, 75%, and 95% v/v ethanol before infiltration of the samples in epoxy resin. Resin blocks were cut in 70-nm sections using Leica UC 7 microtome (Leica) and observed with a Tecnai T12 TEM FEI operated at 120 kV.

### Flow Cytometry Analysis

RAW264.7 cells were plated the day before infection at 0.3 × 10^6^ cells/ml and were incubated overnight at 37°C, 5% CO_2_. For infection, 50% of the media was removed and replaced by bacterial suspension. After infection with CFSE-labeled leptospires and gentamicin-protection assay, the percentage of infected macrophages was assessed by flow cytometry. The cells were scrapped in cell dissociation buffer (Gibco), transferred in U-bottom 96-well plates (TPP), and centrifuged (300 g, 7 min, 4°C). The cell pellet was resuspended in 50 µl of 1:1,000 fixable viability dye efluor780 (eBioscience) in PBS for 10 min on ice. Cells were washed by addition of 150 µl of flow cytometry buffer (Mg^2+^- and Ca^2+^-free PBS, 0.5% v/v FCS, 2 mM EDTA). After another round of centrifugation, the cells were fixed in 50 µl of 4% w/v pFA for 10 min on ice and washed as previously indicated in cytometry buffer. The cells were maintained in cytometry buffer at 4°C before acquisition on CytoFLEX (Beckman Coulter, Brea, CA, USA). After calibration, between 10,000 and 50,000 events were recorded *per* condition and the percentage of infected macrophages, corresponding to $\frac{live\;CFSE^{+}cells}{total\;live\;cells}\times 100$ , was analyzed with FlowJo V10 software.

### Small Interfering RNA Transfection

RAW264.7 cells were plated the day before transfection at 0.1 × 10^6^ and were incubated overnight at 37°C, 5% CO_2_. In each well, transfection was performed using 4.5 µl of Lipofectamine siRNA Max reagent (Fisher Scientific) in 650 µl of OptiMEM media (Gibco) according to the manufacturer’s instructions. The final concentration of predesigned siRNA targeting *atg5* (FlexiTube, Qiagen, Hilden, Germany) was 80 nM, and the corresponding scramble siRNA (all start negative, Qiagen) was performed as control. The siRNA preparations in lipofectamine were incubated at room temperature for 25 min before gentle drop-by-drop addition to the cells. After 12 h, 350 µl of complete RPMI was added in the cells. The infection was performed 24 h post-transfection in complete RPMI. Efficiency of the siRNA was assessed by Western blot to evidence the reduction on Atg5 levels.

### SDS-PAGE and Western Blot

RAW264.7 cells or BMDMs were plated the day before infection at 0.3 × 10^6^ or 1 × 10^6^ cells/ml, respectively, and were incubated overnight at 37°C, 5% CO_2_. For infection, 50% of the media was removed and replaced by bacterial suspension. After infection, cell supernatants were removed, and 1 ml of cell dissociation buffer (Gibco) was added to the adherent cells and incubated for 5 min. Next, cells were gently scrapped and transferred into 1.5 ml tubes for centrifugation at 400 g, for 10 min, at 4°C. Supernatants were discarded, and the pellets were resuspended in 50 µl of ice-cold RIPA lysis buffer supplemented with 1× complete Mini, EDTA-free protease inhibition cocktail (Roche, Basel, Switzerland). Cells were incubated on ice for 20 min to allow complete lysis, followed by a centrifugation step at 12,000 g, for 20 min, at 4°C to separate insoluble material. Supernatants were transferred into a new cold 1.5-ml tube, and non-soluble material was discarded. Proteins were dosed using the Bradford assay and protein concentration adjusted to 1 mg/ml, 4× Laemmli buffer (Bio-Rad, Hercules, CA, USA) added, and the samples denatured at 99°C for 10 min. SDS-PAGE was done using 4-15% gradient acrylamide stain-free gels (Bio-Rad), loaded with 5–10 µg of protein extract *per* lane, and gels were ran at 100–120 V in Tris–glycine–SDS buffer (Bio-Rad). To evaluate the total protein amount loaded, proteins were visualized using stain-free reagent in ChemiDoc (Bio-Rad) using a 5-min exposition time and transferred onto an ethanol-pre-activated 0.22-µm PVDF membrane (Bio-Rad) using the Mixed MW fast transfer program of 1 miniGel (Bio-Rad). The membrane was blocked with 5% w/v BSA in TBS with 0.05% v/v Tween 20 (TBS-T) for 60 min at room temperature with mild agitation. Next, the membrane was probed with either rabbit anti-mouse LC3 or rabbit anti-mouse Atg5 (**Table 1**) in 5% w/v BSA in TBS-T overnight at 4°C. In the case of LC3 staining, the membrane was cut at 35 kDa before primary antibody incubation, as suggested in the current literature to avoid unspecific binding of the antibody (Sharifi et al., 2015). The membranes were then washed in TBS-T three times for 15 min followed by incubation with secondary anti-rabbit IgG HRP-linked (**Table 1**) in 5% w/v BSA in TBS-T for 60 min protected from light at room temperature, followed by three washes in TBS-T for 15 min. Blots were revealed using the Clarity reagent (Bio-Rad) with automatic exposure time.

**Table 1:** Antibodies list for Western blot analyses.

Application*	Target	Clonality**	Conjugated	Host	Dilution	Reference	Supplier
WB	LC3	pAb	Purified	Rabbit	1:1.000	L7543	Sigma
WB	Atg5	mAb	Purified	Rabbit	1:1.000	D5F5U	CST
WB	Rabbit IgG	pAb	HRP	Goat	1:10.000	7074S	CST

*WB, Western blot; **mAb, monoclonal antibody; pAb, polyclonal antibody.

### Production of Reactive Oxygen Species and Nitric Oxide

NO dosage was performed on fresh cellular supernatants 24 h post-infection, using the Griess reaction as previously described (Bonhomme et al., 2020). For ROS production assay, cells were seeded in transparent-bottom black 96-well plates (Greiner) in complete RPMI the day before infection. Before infection, the cells were washed twice in HBSS (Gibco) and the 2′,7′-dichlorofluorescein diacetate probe (DCF, Sigma) was loaded in HBSS at a final concentration of 100 µM for 1 h. After loading, the media were removed, and the cells were infected with leptospires or stimulated with purified leptospiral LPS (Bonhomme and Werts, 2020) in HBSS medium (Gibco). Fluorescence (excitation 500 nm/emission 520 nm) was monitored on a Spark fluorimeter (TECAN) for 3–4 h post-infection (kinetics with measurement every 5–10 min).

### Statistical Analyses

Statistical analyses were performed using Student’s *t*-test, with asterisks corresponding to *p* values: * for *p* < 0.05; ** for *p* < 0.01; *** for *p* < 0.001.

## Results

### Leptospires Are Internalized in Murine Macrophages

Peritoneal macrophages are important for the control of leptospirosis after intra-peritoneal (IP) injection of leptospires (Ferrer et al., 2018; Santecchia et al., 2019), illustrating that phagocytes play a role at the early phase of the disease. Interestingly, after IP injection, bacteria disseminate in blood and subsequently reach target organs such as the liver and kidneys (Ratet et al., 2014). We therefore started our study by addressing the role of phagocytes in the blood stage of leptospirosis, by performing intravenous depletion of patrolling monocytes *via* clodronate injection (**Figure S1A**) in C57BL/6J albino mice. Our experimental setup allowed a specific decrease in blood of the percentage of Ly6C^-^ patrolling monocytes, without alteration of either classical Ly6C^+^ monocytes or neutrophil populations (**Figure S1B, C****)**. We then infected IP either clodronate- or PBS-treated mice with a sublethal dose of bioluminescent *L. interrogans* and observed by live imaging that clodronate-depleted mice had much higher leptospiral burden at the acute and chronic phases of the disease (**Figure S1D**), suggesting that patrolling monocytes also play a role in the control of the bacteria. Taken together, these data reinforce the idea that phagocytes play a role in controlling experimental leptospirosis in both the peritoneum and blood stages in mice.

**Figure 1:**
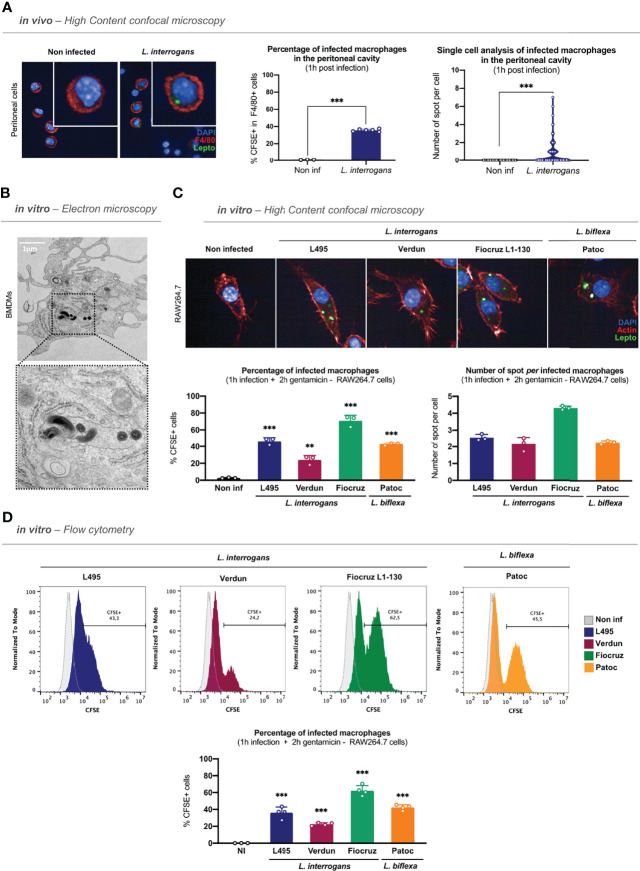
Leptospires are internalized in murine macrophages. **(A)** High-content (HC) confocal microscopy images and analyses of adherent F4/80^+^ peritoneal cells 1 h post intra-peritoneal injection of 5 × 10^7^ CFSE-labeled *L. interrogans* serovar Manilae strain L495 in C57BL/6J mice. **(B)** Electron microscopy images of bone marrow-derived macrophages infected 1 h with MOI 100 of *L. interrogans* serovar Manilae strain L495. **(C)** HC confocal microscopy images and analyses of RAW264.7 cells infected 1 h with MOI 100 of CFSE-labeled *L. interrogans* serovar Manilae strain L495, serovar Copenhageni strain Fiocruz L1–130, serovar Icterohaemorrhagiae strain Verdun, and *L. biflexa* serovar Patoc strain Patoc I and after gentamicin protection assay. **(D)** Flow cytometry histograms and analyses of RAW264.7 cells infected 1 h with MOI 100 of CFSE-labeled *L. interrogans* serovar Manilae strain L495, serovar Copenhageni strain Fiocruz L1–130, serovar Icterohaemorrhagiae strain Verdun, and *L. biflexa* serovar Patoc strain Patoc I and after gentamicin protection assay. **(A–D)** Bars correspond to mean +/- SD of technical replicates (*n* = 3). Data presented are representative of at least two independent experiments.

After confirming the role of phagocytes in the control of leptospirosis, we addressed whether leptospires were internalized in murine macrophages *in vivo*. To track leptospires both *in vivo* and *in vitro*, we fluorescently labeled the bacteria using CFSE, as this stain was previously shown to maintain leptospiral virulence (Liu et al., 2014). After controlling that CFSE staining did not alter the morphology of leptospiral serovars used in this study (**Figure S2A**), we infected intra-peritoneally C57BL/6J mice with 5 × 10^7^ CFSE–labeled leptospires/mouse. One hour post-infection, we performed peritoneal lavages with media containing gentamicin and recovered the peritoneal cells. After adherence of the peritoneal cells and fixation, we positively stained macrophages with anti–F4/80. We first observed that 30% of the adherent cells of the peritoneal cavity were F4/80^+^ macrophages (**Figure S2B**). We then assessed the internalization of *L. interrogans* serovar Manilae strain L495 in F4/80^+^ macrophages by confocal microscopy. Our results showed that CFSE-labeled leptospires can be found as round spots inside peritoneal macrophages 1 h post-infection (**Figure 1A****; left panel**). Using high-content (HC) confocal microscopy, we then quantified the percentage of macrophages positive for fluorescent spots, and we performed single-cell analysis on the number of spots *per* cell. Our results showed that 40% of peritoneal macrophages were infected by leptospires and that they contained between one to eight spots *per* cell (**Figure 1A****; right panels**).

Following these observations, we investigated the internalization of leptospires *in vitro* in either BMDMs or RAW264.7 cells. After 1 h of infection, to remove all extracellular leptospires, we performed washes and a protection assay by incubating the cells for 2 h in gentamicin–containing medium, as detailed in the time frame of the experimental protocol (**Figure S2C**). We first analyzed BMDMs by transmission electron microscopy that showed that spiral-shaped *L. interrogans* serovar Manilae strain L495 can indeed be found intracellularly (**Figure 1B**). We then infected RAW264.7 cells with different serovars of pathogenic *L. interrogans* (serovar Manilae strain L495, serovar Icterohaemorrhagiae strain Verdun, serovar Copenhageni strain Fiocruz L1–130) and with saprophytic *L. biflexa* (serovar Patoc strain Patoc I) and analyzed the cells by HC confocal microscopy 3 h post-infection with CFSE-labeled bacteria. Our results showed that all the serotypes of both *L. interrogans* and *L. biflexa* were internalized in RAW264.7 cells (**Figure 1C****; upper panel)**. Using a reconstructed z-stack of confocal microscopy images, we were able to provide short movies in which CFSE-labeled leptospires are visible within the actin cytoskeleton of the macrophages (**Figure S2D** and **videos**). Interestingly, microscopy quantifications confirmed that 40% of macrophages were infected with the strain L495 (**Figure 1C****; lower left panel**), consistent with the *in vivo* findings. Infection with the Verdun strain resulted in 20% of infected macrophages, whereas 70% of macrophages were infected with Fiocruz, suggesting that although all serotypes were internalized, they did not have the same internalization rate (**Figure 1C****; lower left panel**). Finally, quantification of the number of spots *per* cell revealed an average of two to three spots *per* cell upon infection with the strains L495, Verdun, and Patoc, whereas there were up to four spots *per* cell in the case of the strain Fiocruz (**Figure 1C****, lower right panel**). Using the same infection protocol, we confirmed these findings by flow cytometry analyses. Our results showed populations of macrophages positive for CFSE–labeled leptospires 3 h post-infection with all the serotypes (**Figure 1D****, upper panel and** **Figure S2E**), and we confirmed the internalization percentages observed by microscopy (**Figure 1D****, lower panel**).

### Leptospires Are Also Internalized in Human Macrophages

**Figure 2:**
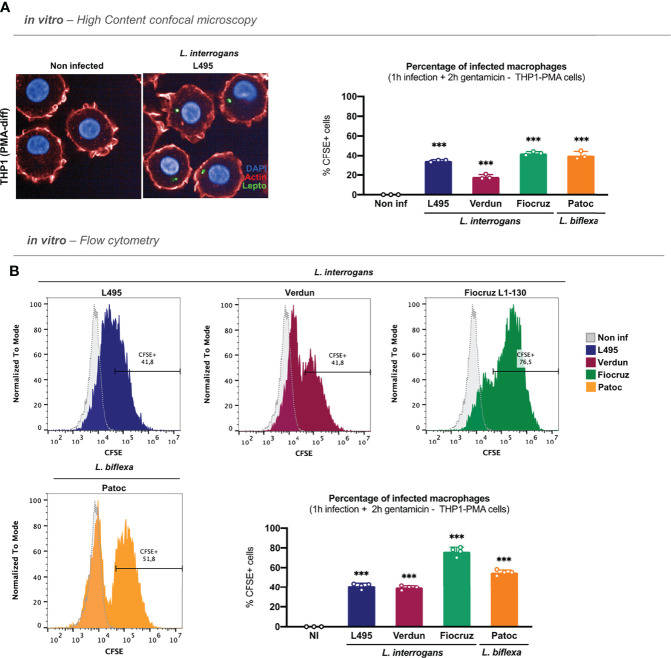
Leptospires are also internalized in human macrophages. **(A)** High-content (HC) confocal microscopy images and analyses of PMA–differentiated THP1 cells infected for 1 h with MOI 100 of CFSE-labeled *L. interrogans* serovar Manilae strain L495, serovar Copenhageni strain Fiocruz L1–130, serovar Icterohaemorrhagiae strain Verdun, and *L. biflexa* serovar Patoc strain Patoc I and after gentamicin protection assay. **(B)** Flow cytometry histograms and analyses of PMA–differentiated THP1 cells infected for 1 h with MOI 100 of CFSE-labeled *L. interrogans* serovar Manilae strain L495, serovar Copenhageni strain Fiocruz L1–130, serovar Icterohaemorrhagiae strain Verdun, and *L. biflexa* serovar Patoc strain Patoc I and after gentamicin protection assay. **(A, B)** Bars correspond to mean +/- SD of technical replicates (*n* = 3). Data presented are representative of at least two independent experiments.

A study reported that leptospires could be internalized differently in human and murine macrophages (Li et al., 2010). To address this question, we infected PMA-differentiated human THP1 cells (THP1-PMA) with CFSE–labeled *L. interrogans* serovar Manilae strain L495 for 1 h and, after gentamicin–protection assay, we analyzed the internalization of leptospires by microscopy. Our results showed that leptospires were also internalized and found as round spots in human macrophages (**Figure 2A****; left panel**). Furthermore, we performed infections with *L. interrogans* serovar Manilae strain L495, serovar Icterohaemorrhagiae strain Verdun, serovar Copenhageni strain Fiocruz L1-130, and *L. biflexa* serovar Patoc strain Patoc I. Likewise in murine macrophages, our results showed that all four serotypes of leptospires were internalized in human macrophages, although the internalization rate differed between strains (**Figure 2A****; right panel**). Here again, infection with the strain Verdun led to a lower internalization rate than the other serotypes (**Figure 2A****; right panel**). However, we did not observe different internalization rates between the strains L495, Fiocruz, and Patoc (**Figure 2A****; right panel**). Flow cytometry analyses performed after gentamicin–protection assay confirmed that internalization with all the serotypes of leptospires triggered the appearance of population(s) of macrophages positive for CFSE–labeled leptospires (**Figure 2B****; upper panel and** **Figure S3**). Our results showed here that the strain Fiocruz was internalized more efficiently than the other strains (**Figure 2B****; lower panel**).

### Internalized Leptospires Do Not Replicate in Macrophages and Disappear Between 3 and 6 h pi

**Figure 3:**
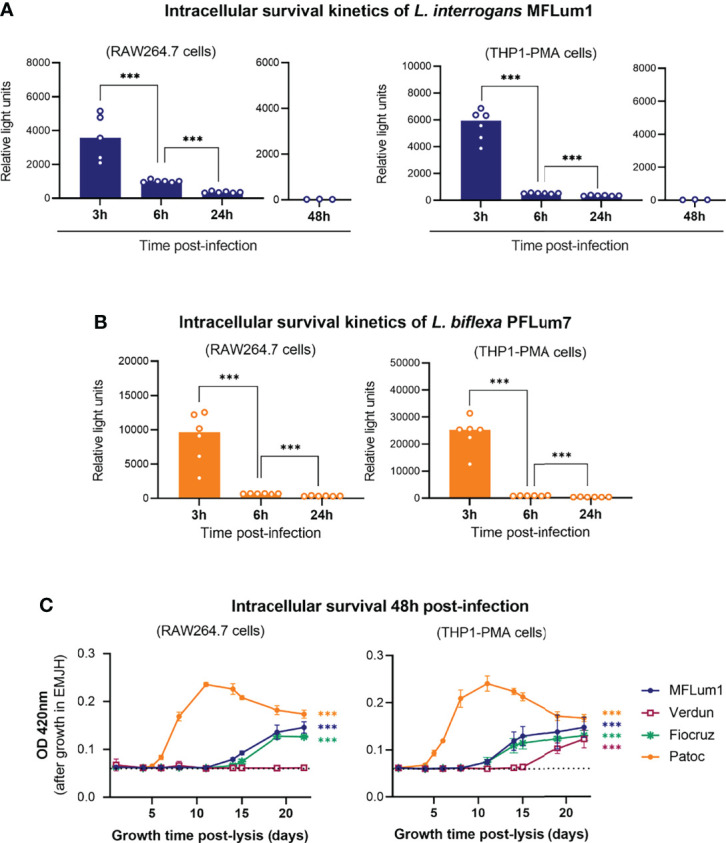
Internalized leptospires do not replicate in macrophages and disappear between 3 and 6 hpi. **(A, B)** Intracellular survival 3, 6, 24, and 48 h post-infection of **(A)** *L. interrogans* serovar Manilae strain MFLum1 and **(B)** *L. biflexa* serovar Patoc strain PFLum in both RAW264.7 and PMA–differentiated THP1 cells infected during 1 h with subsequent gentamicin protection assay. Intracellular survival was directly assessed by luminescence measurement on cell lysates diluted in EMJH. Bars correspond to mean +/- SD of technical replicates (*n* = 6). **(C)** Intracellular survival 48 h post-infection of *L. interrogans* serovar Manilae strain MFLum1, serovar Copenhageni strain Fiocruz L1–130, serovar Icterohaemorrhagiae strain Verdun, and *L. biflexa* serovar Patoc strain Patoc I in both RAW264.7 and PMA–differentiated THP1 cells infected during 1 h with subsequent gentamicin protection assay. Intracellular survival was indirectly measured by monitoring leptospiral growth from cell lysates diluted in EMJH at OD 420 nm. Dots correspond to mean +/- SD of technical replicates (*n*=3). Data presented are representative of at least 3 independent experiments.

A study reported that leptospires can replicate in human macrophages, but not in murine macrophages (Li et al., 2010). However, another study reported conflicting results (Zhao et al., 2013). Other studies also reported a distinct fate for pathogenic and saprophytic leptospires (Merien et al., 1997; Toma et al., 2011). We therefore investigated the intracellular survival kinetics of pathogenic and saprophytic leptospires in both cell types. First, we infected RAW264.7 and THP1-PMA cell lines with bioluminescent *L. interrogans* serovar Manilae strain MFLum1 (L495 derivative) (Ratet et al., 2014) and performed a 1 h infection followed by the gentamicin-protection assay. We then lysed the macrophages at 3, 6, 24, and 48 h and measured the luminescence, which is proportional to the amount of live intracellular leptospires (Ratet et al., 2014). Of note, the luminescence intensity is higher for the more metabolically active *L. biflexa* strain PFLum7 than for the *L. interrogans* strain MFLum1. Absolute luminescence levels can therefore not be compared between the two strains. Our results showed that live pathogenic MFLum1 were found intracellularly at 3 h post-infection in both RAW264.7 and THP1–PMA cells. In addition, we observed a drastic decrease in the luminescence at 6 h and even more at 24 and 48 h post-infection (**Figure 3A**). The results obtained 48 h post-infection suggest that leptospires do not replicate in macrophages, since the average generation time for this strain is around 18 h (Ratet et al., 2014). Importantly, we excluded that this decrease in luminescence could be associated with toxicity of the culture medium (**Figure S4A**) or that the intracellular leptospires could be directly killed by gentamycin (**Figure S4B**). We then performed the same experiment using the bioluminescent saprophytic *L. biflexa* serovar Patoc strain PFLum7 (Patoc 1 derivative) (Ratet et al., 2014). Our results showed that PFLum7–associated luminescence was also measured at 3 h post-infection in both cell types. We observed the same significant decrease in intracellular luminescence at 6 and 24 h post-infection (**Figure 3B**). As the saprophytic strain has a lower generation time of around 4 h, the decrease observed at 24 h is enough to conclude that *L. biflexa* do not replicate in macrophages. To address the intracellular survival of other strains of *L. interrogans* for which we did not have bioluminescent derivatives (serovar Icterohaemorrhagiae strain Verdun and serovar Copenhageni strain Fiocruz L1-130), we used the same protocol of infection and gentamicin assay but inoculated the lysates in EMJH medium. We then measured optical density (OD) at 420 nm several days post-infection (depending on the generation time of the strain). We assumed that the growth of the culture would be proportional to the inoculate and would therefore allow us to indirectly assess the intracellular survival kinetics of these strains. Our results showed that, like for MFLum1 and PFLum7, the intracellular leptospiral loads of strains Verdun and Fiocruz decreased in a time-dependent manner in both RAW264.7 and THP1–PMA cells (**Figure S4C**). Overall, these results suggest that leptospires do not replicate inside either murine or human macrophages.

Although the majority of the leptospires did not seem to remain intracellular, we further decided to investigate the potential survival of minor populations of leptospires at a longer time point. To do so, we infected both mouse and human macrophages with all the different serotypes of leptospires and lysed the cells 48 h post-infection. Because the leptospiral loads were undetectable by luminescence, we inoculated the macrophage lysates and monitored OD as indirect measurement of intracellular survival. Our results revealed leptospiral growth in almost all the conditions, suggesting that minor populations of leptospires were able to survive up to 48 h post-infection (**Figure 3C**). Interestingly, no leptospiral culture was evidenced from the lysate of RAW264.7 cells lysed 48 h post-infection with Verdun strain (**Figure 3C**). Of note, the different generation time of the strains (*L. biflexa* Patoc: 4 h, pathogenic *L. interrogans*: 18–24 h) may explain the different slope of the growth curves.

### Internalized Leptospires Are Not Degraded by Lysosomal-Dependent Pathways

**Figure 4:**
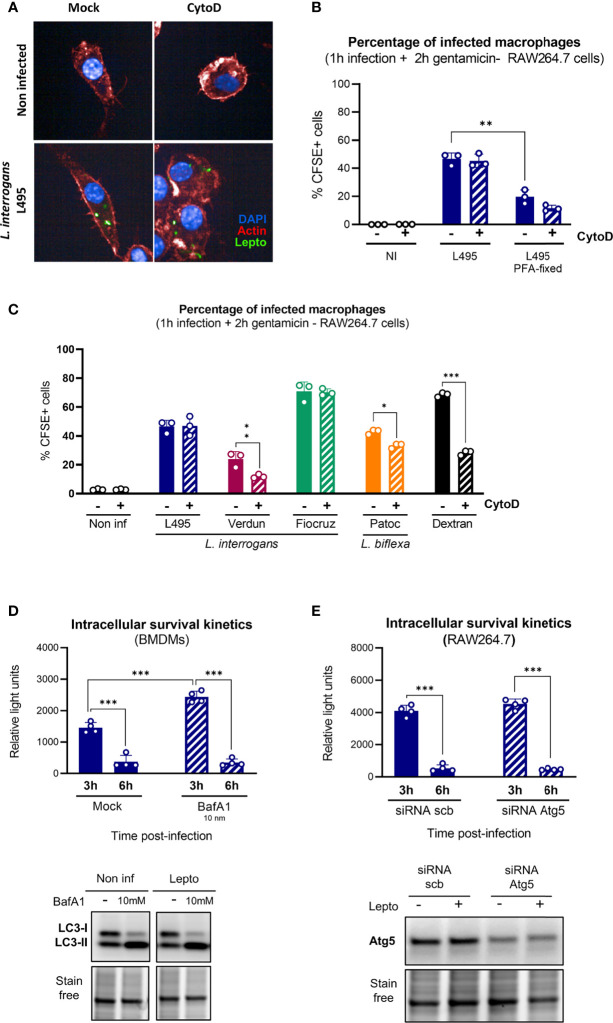
Internalized leptospires are not degraded by lysosomal pathways. **(A, B)** High-content (HC) confocal microscopy **(A)** images and **(B)** analysis of RAW264.7 cells treated with Cyto D, infected for 1 h with MOI 100 of live or *p*FA–fixed CFSE-labeled *L. interrogans* serovar Manilae strain L495 and after gentamicin protection assay. **(C)** HC confocal microscopy analysis of RAW264.7 cells treated with Cyto D, infected for 1 h with MOI 100 of CFSE-labeled *L. interrogans* serovar Manilae strain L495, serovar Copenhageni strain Fiocruz L1–130, serovar Icterohaemorrhagiae strain Verdun, or *L. biflexa* serovar Patoc strain Patoc I and after gentamicin protection assay. **(D)** Intracellular survival 3 and 6 h post-infection of *L. interrogans* serovar Manilae strain MFLum1 in BMDMs pretreated with 10 mM BafA1 and infected during 1 h with subsequent gentamicin protection assay. Intracellular survival was directly assessed by luminescence measurement on cell lysates diluted in EMJH. WB analysis of LC3 in BMDMs cells 3 h post-infection in the same conditions. **(E)** Intracellular survival 3 and 6 h post-infection of *L. interrogans* serovar Manilae strain MFLum1 in RAW264.7 transfected with scramble or Atg5 siRNA and infected during 1 h with subsequent gentamicin protection assay. Intracellular survival was directly assessed by luminescence measurement on cell lysates diluted in EMJH. WB analysis of Atg5 in RAW264.7 cells 3 h post-infection in the same conditions. **(A–E)** Bars correspond to mean +/- SD of technical replicates (*n* > 3). Data presented are representative of at least two independent experiments.

Macrophages are phagocytic cells that have the capacity to deliver pathogens into the lysosomal pathway for killing and degradation. These cells have evolved different ways for lysosomal delivery, among which are phagocytosis, endocytosis, and autophagy. Evidence in the literature suggests that non–opsonized leptospires are not phagocytosed by macrophages (Santecchia et al., 2020). Considering that our results showed that intracellular leptospires disappear between 3 and 6 h post-infection led us to reevaluate the contribution of phagocytosis upon infection by leptospires. To address this question, we infected RAW264.7 cells pretreated with the actin polymerization inhibitor cytochalasin D (Cyto D). Microscopy analyses performed after gentamicin assay revealed that *L. interrogans* strain L495 was still found inside macrophages upon CytoD treatment (**Figure 4A**). We performed HC microscopy quantifications, which revealed no differences in the infection percentage with or without blocking actin polymerization (**Figure 4B**). This result suggests that the L495 strain actively infects macrophages, independently of phagocytosis. To confirm this hypothesis, we infected RAW264.7 with *para*formaldehyde (pFA)-fixed leptospires strain L495, which were equivalently labeled with CFSE compared to live bacteria. Although infection with live L495 resulted in 45% infected macrophages, we observed less than 20% of macrophages with inactivated leptospires (**Figure 4B**), suggesting again that leptospires enter actively in murine macrophages. To address the contribution of actin polymerization in the internalization of other serotypes of leptospires, we also infected CytoD-pretreated RAW264.7 cells with *L. interrogans* serovar Icterohaemorrhagiae strain Verdun, serovar Copenhageni strain Fiocruz L1–130, and saprophytic *L. biflexa* serovar Patoc strain Patoc I, in addition to FITC-labeled dextran beads as control of CytoD activity. Our results showed that CytoD did not affect either the internalization of the Fiocruz strain (**Figure 4C**). However, Verdun and Patoc strains showed a slightly reduced internalization rate when actin polymerization was inhibited (**Figure 4C**), suggesting that only a small population of these leptospiral strains could be phagocytosed.

We then hypothesized that other lysosome–dependent pathways such as LC3–associated phagocytosis (LAP) or autophagy could explain the leptospiral intracellular fate. We first used bafilomycin A1 (BafA1), which prevents the acidification of lysosomes and consequent cargo degradation, to study the contribution of lysosome–dependent pathways. We evaluated intracellular *L. interrogans* strain L495 loads after infection and gentamicin protection assay of RAW264.7 treated or not with 10 nM BafA1 cells. First, we observed an increase in the amount of internalized leptospires 3 h post-infection upon BafA1 treatment (**Figure 4D****, upper panel**). However, the observed reduction in intracellular leptospiral load between 3 and 6 h was similar in both non–treated and BafA1 conditions (**Figure 4D****, upper panel**). These results suggested that, although lysosome–dependent pathways could play a role in the early control of internalized leptospires, they are not the main reason for the major reduction in bacterial loads observed between 3 and 6 h. The efficiency of BafA1 was controlled by monitoring the turnover of LC3–II (an autophagic protein known to be recycled in a lysosome-dependent manner). Indeed, upon autophagy induction, LC3-I gets lipidated to form LC3-II that spans the autophagosome double membrane. Blocking the autophagy flux using BafA1 must result in an accumulation of undegraded autophagosomes and consequently increase the LC3-II protein levels in the cytosol. Our results confirmed the expected LC3–II accumulation upon BafA1 treatment (**Figure 4D****, lower panel**).

Next, to confirm specifically that autophagy and LAP were not responsible for the clearance of intracellular leptospires, we used siRNA targeting the autophagy-related protein Atg5, essential for canonical autophagosome nucleation (Mizushima et al., 1998). After infection with *L. interrogans* strain L495 and gentamicin-protection assay, we observed no difference between control and *atg5* siRNA conditions and witnessed the same reduction in leptospiral loads between 3 and 6 h (**Figure 4E****, upper panel**). Efficient reduction of Atg5 proteins levels were confirmed by WB analyses (**Figure 4E****, lower panel**). These results confirmed that LAP and Atg-dependent autophagy play no role in the control of intracellular leptospires.

### Disappearance of Leptospires Between 3 and 6 h pi Is Not TLR-Mediated

**Figure 5:**
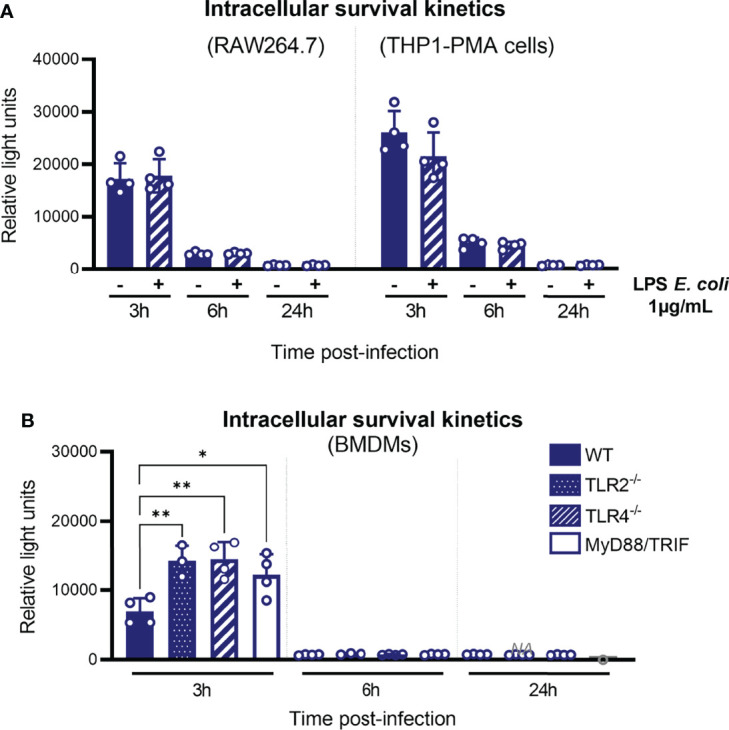
Disappearance of leptospires between 3 and 6 hpi is not TLR-mediated. **(A)** Intracellular survival 3, 6, and 24 h post-infection of *L. interrogans* serovar Manilae strain MFLum1 in RAW264.7 stimulated with 1 µg/ml LPS of *E coli* and infected during 1 h with subsequent gentamicin protection assay. Intracellular survival was directly assessed by luminescence measurement on cell lysates diluted in EMJH. **(B)** Intracellular survival 3, 6, and 24 h post-infection of *L. interrogans* serovar Manilae strain MFLum1 in WT, TLR2 ko, TLR4 ko, and MyD88/TRIF dko BMDMs infected during 1 h with subsequent gentamicin protection assay. Intracellular survival was directly assessed by luminescence measurement on cell lysates diluted in EMJH. **(A, B)** Bars correspond to mean +/- SD of technical replicates (*n*>3). Data presented are representative of at least two independent experiments.

TLR2 and TLR4 are important for the control of leptospires in mice (Chassin et al., 2009). Furthermore, a study suggested that murine macrophages, but not human, can control intracellular leptospires (Li et al., 2010). Considering that the leptospiral LPS activates murine TLR4, but not human TLR4 (Nahori et al., 2005), it was therefore hypothesized that LPS sensing through TLR4 could mediate the control of leptospires in murine cells. Although our results reevaluated such conclusions and showed no difference in both cell types (**Figures 1****, 2**), we believed important to investigate the potential role of TLRs in the intracellular fate of leptospires. First, we addressed specifically the role of LPS signaling through TLR4 in controlling intracellular leptospires. To do so, we assessed the kinetics of the intracellular survival of strain MFLum1 by performing gentamicin-protection assay in mouse and human macrophages pre-stimulated with *E. coli* LPS, a potent TLR4 agonist (**Figure 5A**). We observed a time-dependent reduction in the intracellular leptospiral loads in both human and murine cell lines, with no difference either pretreated with LPS or not (**Figure 5A**). These results suggest that TLR4 activation is not responsible for the control of leptospires in either mouse or human cells.

Next, to investigate more generally the role of TLRs in the control of intracellular leptospires, we infected BMDMs from WT and TLR2, TLR4, and MyD88/TRIF double-KO (DKO) mice with *L. interrogans* strain MFLum1 and performed gentamicin assay. We observed an increase in the amount of internalized leptospires 3 h post-infection in all the KO BMDMs (**Figure 5B**). However, at 6 and 24 h post-infection macrophages from all the different genotypes showed a decrease in internalized leptospires in a similar way (**Figure 5B**). This suggests that, although TLRs could play a role early upon infection by leptospires, the disappearance phenotype observed between 3 and 6 h post-infection is independent of TLRs and their signaling.

### Internalized Leptospires Are Not Degraded by ROS or NO

**Figure 6:**
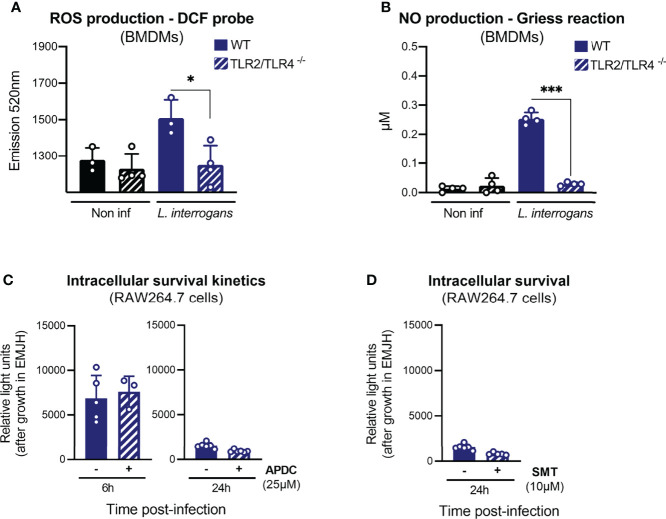
Internalized leptospires are not degraded by ROS or NO. **(A)** ROS production, measured by DCF probe, in WT and TLR2/TLR4 dko BMDMs upon 6 h of infection with *L. interrogans* serovar Manilae strain L495. **(B)** NO production, measured by Griess reaction, in WT and TLR2/TLR4 dko BMDMs upon 24 h infection with *L. interrogans* serovar Manilae strain L495. **(C)** Intracellular survival 6 and 24 h post-infection of *L. interrogans* serovar Manilae strain MFLum1 in RAW264.7 treated with 25 µM ROS-inhibitor APDC and infected during 1 h with subsequent gentamicin protection assay. Intracellular survival was directly assessed by luminescence measurement on cell lysates diluted in EMJH. **(D)** Intracellular survival 24 h post-infection of *L. interrogans* serovar Manilae strain MFLum1 in RAW264.7 treated with 10 µM NO-inhibitor SMT and infected during 1 h with subsequent gentamicin protection assay. Intracellular survival was directly assessed by luminescence measurement on cell lysates diluted in EMJH. **(A–D)** Bars correspond to mean +/- SD of technical replicates (*n* > 3). Data presented are representative of at least two independent experiments.

It has been reported (Li et al., 2017) that ROS are responsible for the killing of intracellular leptospires. However, given our previous results showing that TLRs are not implicated in the fate of internalized leptospires, we decided to reevaluate the contribution of ROS and RNS to the survival of internalized leptospires. We first addressed the contribution of TLR2/TLR4 to the production of these antimicrobial compounds upon infection with leptospires. To do so, we infected WT and TLR2/4 DKO BMDMs with *L. interrogans* strain MFLum1. We observed that leptospires triggered the production of ROS 6 h post-infection and that it was dependent on TLR2/TLR4 (**Figure 6A**). Similarly, production of nitric oxide (NO) 24 h post-infection was also dependent on the presence of TLR2 and TLR4 (**Figure 6B**). We then infected RAW264.7 cells pretreated with APDC, a NOX inhibitor, that efficiently prevented ROS production 6 h post-infection with leptospires (**Figure S5A**). We then performed gentamicin-protection assay, and our results showed that inhibition of ROS production had no impact on the number of internalized leptospires at 6 and 24 h post-infection (**Figure 6C**). We observed that treatment of leptospires with the ROS inhibitor APDC was not toxic for the bacteria (**Figure S5B**). Next, we evaluated the contribution of NO to the phenotype using SMT, a specific inhibitor of iNOS, the inducible enzyme producing NO. Since NO was detected at quantifiable levels in the supernatant of infected cells at 24 h post-infection, we evaluated the survival at this timepoint. We first confirmed the inhibition of NO production by SMT (**Figure S5A**). Our results further evidenced no difference on the internalized leptospires with or without SMT 24 h post-infection (**Figure 6D**). These findings suggest that these two potent antimicrobial compounds do not play a role in the control of intracellular leptospires at 6 and 24 h post-infection.

### Internalized Leptospires Could Exit Both Human and Murine Macrophages

**Figure 7:**
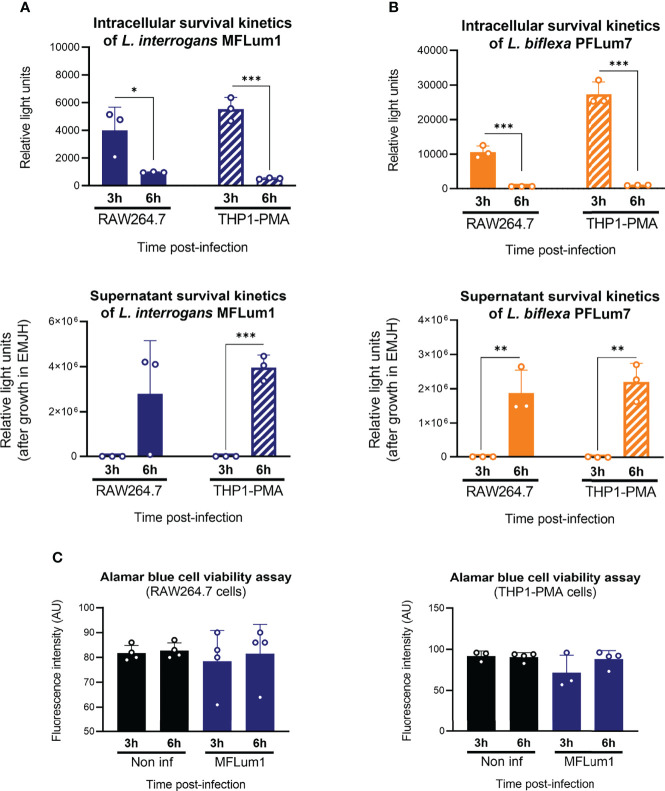
Internalized leptospires seem to exit both human and murine macrophages. **(A, B)** Intracellular and supernatant survival 3 and 6 h post-infection of **(A)** *L. interrogans* serovar Manilae strain MFLum1 and **(B)** *L. biflexa* serovar Patoc strain Patoc I in RAW264.7 and THP1–PMA cells infected during 1 h with subsequent gentamicin protection assay. Intracellular survival was directly assessed by luminescence measurement on cell lysates diluted in EMJH. Supernatant survival was indirectly measured by monitoring leptospiral growth from cell lysates diluted in EMJH at OD 420 nm. **(C)** RAW264.7 and THP1–PMA cell viability measured by Alamar Blue assay in the same infection conditions. **(A–C)** Bars correspond to mean +/- SD of technical replicates (*n* > 3). Data presented are representative of at least two independent experiments.

Our results suggested that the main microbicidal mechanisms of macrophages were not responsible for the reduction in the intracellular leptospiral load after 3 h of infection. In addition, we showed that leptospires actively entered in cells (**Figure 3B**). Given these observations, we hypothesized that leptospires could also exit macrophages. To test such hypothesis, we infected mouse and human macrophages with both pathogenic *L. interrogans* MFLum1 and saprophytic *L. biflexa* PFLum7. At the end of the gentamicin assay protocol, and right before cell lysis and luminescence measurements, we also collected the cell supernatants and inoculated them in EMJH medium to indirectly assess the presence of extracellular leptospires. Direct measurements of the cell lysates revealed again a decrease in luminescence from 3 to 6 h post-infection **(** **Figures 7A, B****, upper panels**). Furthermore, indirect monitoring of the cell supernatants (too diluted to be measured directly) showed no extracellular leptospires at the end of the gentamicin assay 3 h post-infection. Surprisingly, 6 h post-infection, our results revealed the presence of extracellular leptospires in the supernatants of both human and murine macrophages (**Figures 7A, B****; lower panels**). Interestingly, this phenotype was conserved between pathogenic *L. interrogans* and saprophytic *L. biflexa*. While performing the infections, we measured macrophage viability by Alamar Blue which showed no cell death and hence showing that the release of leptospires in the supernatant was not a consequence of macrophage death (**Figure 7C**). Overall, these results showed that the reduction in intracellular leptospiral load between 3 and 6 h post-infection was associated with an increase in extracellular leptospires, suggesting that some leptospires could exit macrophages within this time frame.

## Discussion

Overall, our results showed that pathogenic and saprophytic fluorescently labeled leptospires were internalized in both human and murine macrophages. Using luminescent strains, we further observed a drastic reduction in intracellular leptospiral load between 3 and 24 h, showing that leptospires did not replicate intracellularly. We excluded a major role of lysosomal–dependent pathways (phagocytosis and autophagy), and we further excluded a role for TLRs and TLR–induced ROS and RNS in this phenotype. Our results showed that the reduction in the intracellular leptospiral load was associated with an increase in extracellular leptospires, suggesting that both *L. interrogans* and *L. biflexa* can exit from human and murine macrophages. Finally, results obtained *in vitro* may have a relevance in the host, as we also showed that leptospires are also found intracellularly *in vivo* in peritoneal macrophages upon IP infection in mice.

We have recently reviewed the controversial role of macrophages in leptospirosis (Santecchia et al., 2020). Therefore, our main goal was to reevaluate the internalization of *L. interrogans* and *L. biflexa* using gentamicin–protection assay to unambiguously address the fate of leptospires inside murine and human macrophages. To do so, we first coupled gentamicin–protection assay with sensitive techniques such as flow cytometry and HC confocal microscopy. These techniques allowed us to quantify the percentage of leptospires internalized. Interestingly, we observed 40% of macrophages infected with *L. interrogans* serovar Manilae strain L495 upon both intraperitoneal infection of C57BL/6J mice and infection of RAW264.7 cells cultured in medium supplemented with heat-inactivated serum. These findings are consistent with the leptospiral inoculum used in both protocols, roughly comparable to a MOI 100 (leptospires/macrophages), showing that leptospires can infect macrophages to a similar extent in different experimental setups. Consistent with a previous study that demonstrated *in vivo* internalization of leptospires in zebrafish phagocytes (Davis et al., 2009), these findings reinforce the physiological relevance to study the internalization of leptospires in macrophages. Furthermore, the use of the bioluminescent strains MFLum1 and PFLum7 provided direct sensitive insight on the intracellular fate of leptospires. Indeed, intracellular leptospires were previously quantified using PCR targeting DNA (Toma et al., 2011) that did not allow discrimination between live and dead leptospires, or by indirect colony-forming unit (CFU) assay. Unlike what was reported in other studies (Li et al., 2010; Toma et al., 2011), our results first showed that leptospires did not replicate inside either human or murine macrophages. Although we did not address the specific subcellular localization of leptospires, we showed that the intracellular loads of live *L. interrogans* and *L. biflexa* decreased drastically between 3 and 6 h in both cell types. These results are consistent with results showing a diminution of total cell–associated leptospiral DNA amount between 2 and 24 h, for both pathogenic and saprophytic leptospires (Toma et al., 2011).

Contrary to previous studies (Zhao et al., 2013; Li et al., 2017), we also demonstrated that lysosomal pathways, as well as TLR–dependent ROS and RNS, played no role in this major reduction in bacterial loads observed between 3 and 6 h. However, we cannot exclude that these pathways could have a role at earlier time points. Indeed, although it provides unequivocal results on the internalization of leptospires, the main drawback of the gentamicin–protection assay is that it prevents analysis at early time points post-infection. Our experimental setup included a 1-h infection followed by a 2-h gentamicin treatment, making the first analysis possible only 3 h post-infection. In fact, we observed a slightly higher leptospiral load 3 h post-infection in BafA1-treated cells. Considering that this finding was not reproduced upon CytoD or Atg5 siRNA treatment, we excluded a role of either phagocytosis or autophagy, and we hypothesize that endocytosis could play a small role at early infection time points. Such hypothesis is supported by a study reporting colocalization of *L. interrogans* and *L. biflexa* with early endosome antigen 1 (EEA1), late endosomal antigen 1 (LAMP1), and lysosomal protease cathepsin D (Toma et al., 2011). We also cannot exclude that endocytosis could occur differently upon infection with *L. interrogans* and *L. biflexa*, as it has been suggested (Toma et al., 2011). We observed similar higher leptospiral loads at 3 h post-infection in experiments using TLRs KO compared to WT mice. Such results are consistent with the role of ROS in leptospiral control previously described in murine macrophages (Li et al., 2017). Furthermore, the potential role of TLR4 internalization and TRIF responses, which are avoided by the leptospiral LPS (Bonhomme et al., 2020), requires further investigation. Overall, we believe that the mechanisms described beforehand in the literature (Toma et al., 2011; Li et al., 2017) could account for the small differences observed early on, but our results suggest that they did not contribute to the major reduction of leptospiral load observed between 3 and 6 h post-infection.

Our results showed that the reduction in leptospiral load was not associated with either ROS, RNS or to any other TLR-mediated mechanism. However, these findings only relate to intracellular leptospires. Indeed, when studying the general course of leptospirosis, TLR2 and TLR4 play a key role in the control of *L. interrogans* (Chassin et al., 2009). Furthermore, both IP and IV chlodronate depletion before infection resulted in higher leptospiral burden during both acute and chronic leptospirosis (Ferrer et al., 2018; Santecchia et al., 2019), showing that phagocytes, among which are patrolling monocytes and peritoneal macrophages, play a role in the overall control of IP-injected experimental leptospirosis. Consequently, the potential role for ROS, RNS, and other TLR–mediated mechanism such as antimicrobial peptides on the killing of extracellular leptospires by macrophages is currently under investigation. Taken all together, these studies show that leptospires only transiently reside within cells and that macrophages contribute to the control of the infection, most probably by targeting extracellular leptospires. Henceforth, it is unlikely that leptospires could reside and be transported as cargo inside murine macrophages, as it was previously suggested.

Although leptospires seem to exit the cells before 24 h post-infection, our results showed that viable leptospires could still be recovered after 48 h in macrophages. These findings are consistent with another study that also found *L. interrogans* in cells at late time points (Toma et al., 2011). We cannot exclude that this result could be linked to our experimental protocol. However, the recovery of leptospires 48 h post-infection is proportional to the infection levels at early time points, suggesting that a minor population of leptospires could survive in macrophages. Interestingly, and contrary to other studies (Li et al., 2010; Li et al., 2017), only a minority of leptospires was recovered in both human and murine macrophages, and for both pathogenic and saprophytic leptospires. These results lead us to refute the hypothesis that differential intracellular survival in macrophages could explain the different sensitivity between human and murine hosts (Li et al., 2010). Whether this small population of leptospires surviving intracellularly in a small number of macrophages could play a role in the course of the infection remains to be investigated.

Most intracellular leptospires disappear between 3 and 6 h post-infection, a time frame during which antigen presentation (AP) could be triggered. AP is traditionally associated with phagocytosis (Underhill and Goodridge, 2012) or autophagy (Lee et al., 2010), and our results showing that leptospires escape both pathways led us to hypothesize that they consequently may escape some AP mechanisms. Consistently, it was described that the humoral response to leptospires that confers short-time serovar-specific protection was mostly mediated by their immunogenic LPS, independently of T cells, and therefore independently of antigen presentation to T cells (Chassin et al., 2009; Adler, 2015; Vernel-Pauillac and Werts, 2018). We also showed that *L. interrogans* serovar Icterohaemorrhagiae strain Verdun was less internalized that the other strains and survived less at late time points. Interestingly, this strain had previously been shown to induce a much lower humoral response than the other leptospiral strains (Vernel-Pauillac et al., 2021).

On the other hand, AP can also be triggered after endocytosis (Burgdorf and Kurts, 2008), and our results, taken together with other studies (Toma et al., 2011), suggested that a small population of leptospires could be endocytosed and therefore potentially be presented in the MHC-II context. Hence, the mechanisms underlying potential AP of leptospiral antigens remain to be investigated and could bring novel highlights to design efficient leptospiral long lasting vaccines.

Finally, our results showed that both *L. interrogans* and *L. biflexa* were found in the supernatant and therefore could exit macrophages without inducing cell lysis. A previous study had described similar findings for *L. interrogans*, but not for *L. biflexa* that was never found in the supernatant by counting using the Petroff–Hauser chamber under dark–field microscopy (Toma et al., 2011). We believe that the use of the luminescent strain PFLum7 provided a more sensitive readout allowing to monitor extracellular *L. biflexa* and therefore letting us conciliate these findings. However, a caveat of our protocol is that we could not directly measure by luminescence the bacterial load in the supernatant, because of the large volume and color of medium. Thus, we had to inoculate the supernatant in EMJH medium and wait for several days to measure the luminescence as a proxy of the initial presence of viable leptospires. Therefore, this protocol did not allow us to evaluate the proportion of leptospires found inside and outside, which would have been insightful.

The mechanism by which leptospires can exit the macrophages without cell lysis remains to be investigated. It would be interesting to test if leptospires could exit through vomocytosis, also known as non–lytic expulsion. Historically described after infection with *Cryptococcus neoformans* and *Candida albicans* (Smith and May, 2013), vomocytosis is considered as a mechanism of escape from macrophage killing (Smith and May, 2013). Non–lytic expulsion was also described for *Mycobacterium tuberculosis* and *Legionella pneumophila* (Flieger et al., 2018), therefore opening the question of vomocytosis upon bacterial infection. Overall, our results gave new insights on the fate of intracellular leptospires in macrophages and reevaluated findings of previous studies. We favor a mechanism in which leptospires could be massively exiting the macrophages between 3 and 6 h post-infection, without cell lysis, and the characteristics of this exit are currently under investigation.

## Data Availability Statement

The raw data supporting the conclusions of this article will be made available by the authors, without undue reservation.

## Ethics Statement

The animal study was reviewed and approved by Institut Pasteur ethics committee for animal experimentation (Comité d’Ethique en Expérimentation Animale CETEA registered under #89) and was authorized under #8562 by the French Ministry of Research, the French Competent Authority.

## Author Contributions

Conception, administration, and supervision of the project: CW. Investigation: IS, DB, SP, and CW. Methodology: IS, DB, AG-G, PE, and MM-N. Data analysis: IS, DB, SP, CW, AG-G, and MM-N. Visualization: DB. Validation: IS, DB, SP, and CW. Resources: FV-P. Funding acquisition: CW and IB. DB and IS wrote the original draft, under CW’s supervision. All authors contributed to the review and editing of the manuscript and approved the submitted version.

## Funding

This work was founded by Institut Pasteur grants PTR2017-66 to CW and PTR2019-310 to Nadia Benaroudj (Institut Pasteur, Unité de Biologie des Spirochètes) for AGG salary. IS has been part of the Pasteur-Paris University (PPU) International PhD program. This program has received funding from the Institut Carnot Pasteur Microbes & Santé, and the European Union’s Horizon 2020 research and innovation program under the Marie Sklodowska-Curie grant agreement no. 665807. IS has additionally benefited from a scholarship “Fin de thèse de science” number FDT201805005258 granted by “Fondation pour la recherche médicale (FRM).” This work was supported by Investissement d’Avenir program, Laboratoire d’Excellence “Integrative Biology of Emerging Infectious Diseases” (ANR-10-LABX-62-IBEID) to IB.

## Conflict of Interest

The authors declare that the research was conducted in the absence of any commercial or financial relationships that could be construed as a potential conflict of interest.

## Publisher’s Note

All claims expressed in this article are solely those of the authors and do not necessarily represent those of their affiliated organizations, or those of the publisher, the editors and the reviewers. Any product that may be evaluated in this article, or claim that may be made by its manufacturer, is not guaranteed or endorsed by the publisher.
