# Supplementary figures and images for: Alive Pathogenic and Saprophytic Leptospires Enter and Exit Human and Mouse Macrophages With No Intracellular Replication
Source: Front Cell Infect Microbiol. 2022 Jul 11;12:936931. doi: 10.3389/fcimb.2022.936931 (PMC9310662; doi:10.3389/fcimb.2022.936931)

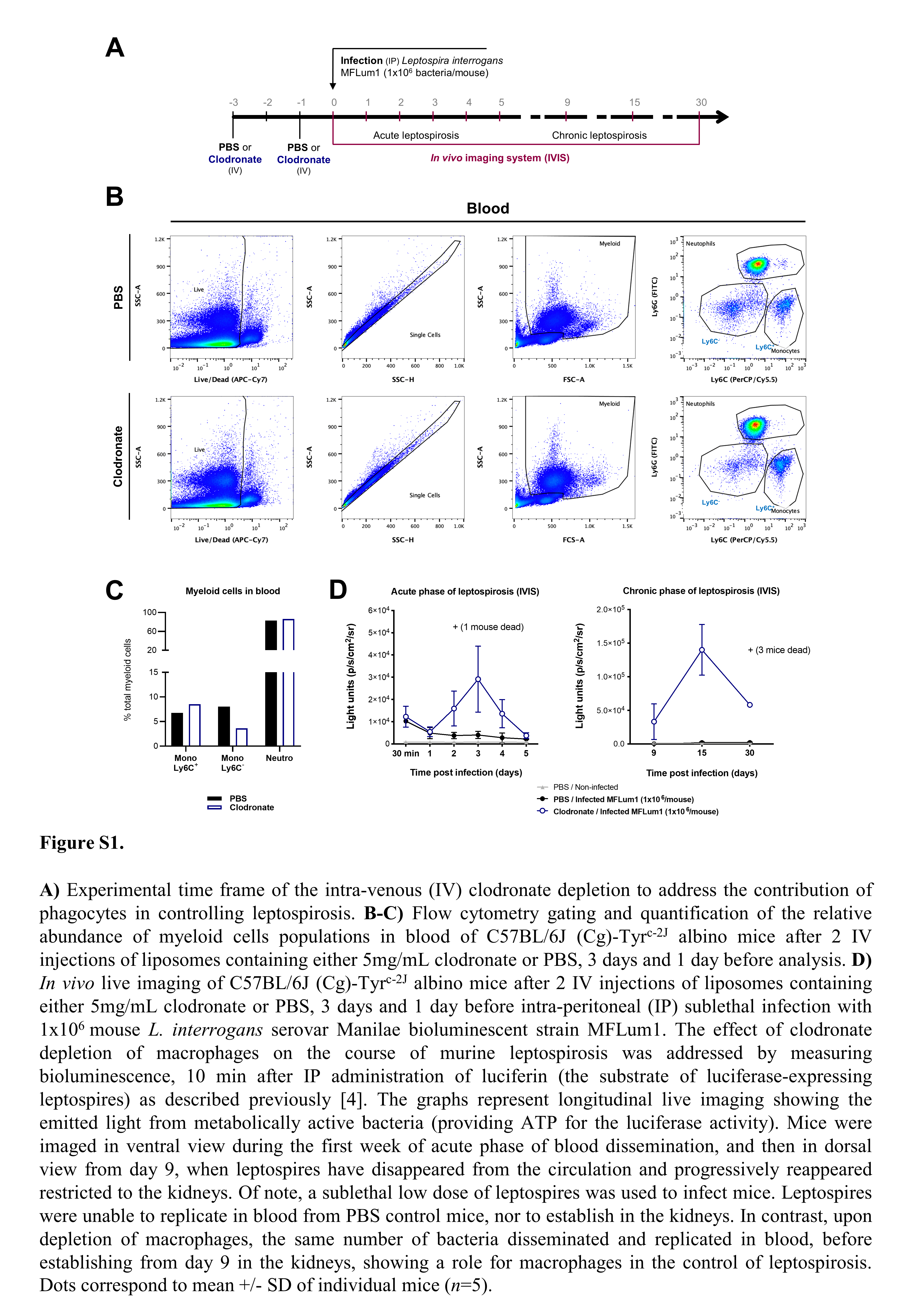

Supplement: Supplementary file 1 [file Image_1.tif]

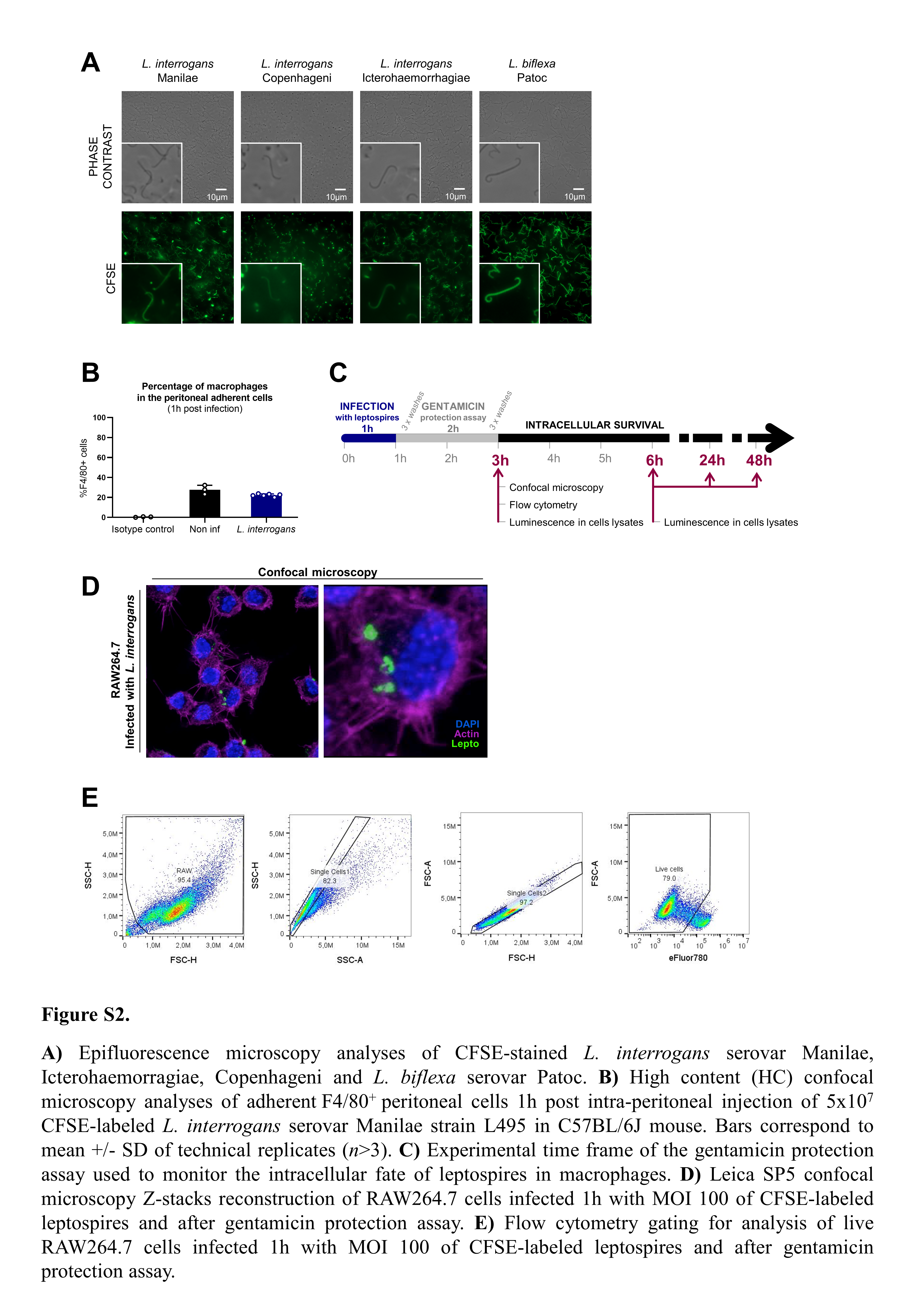

Supplement: Supplementary file 2 [file Image_2.tif]

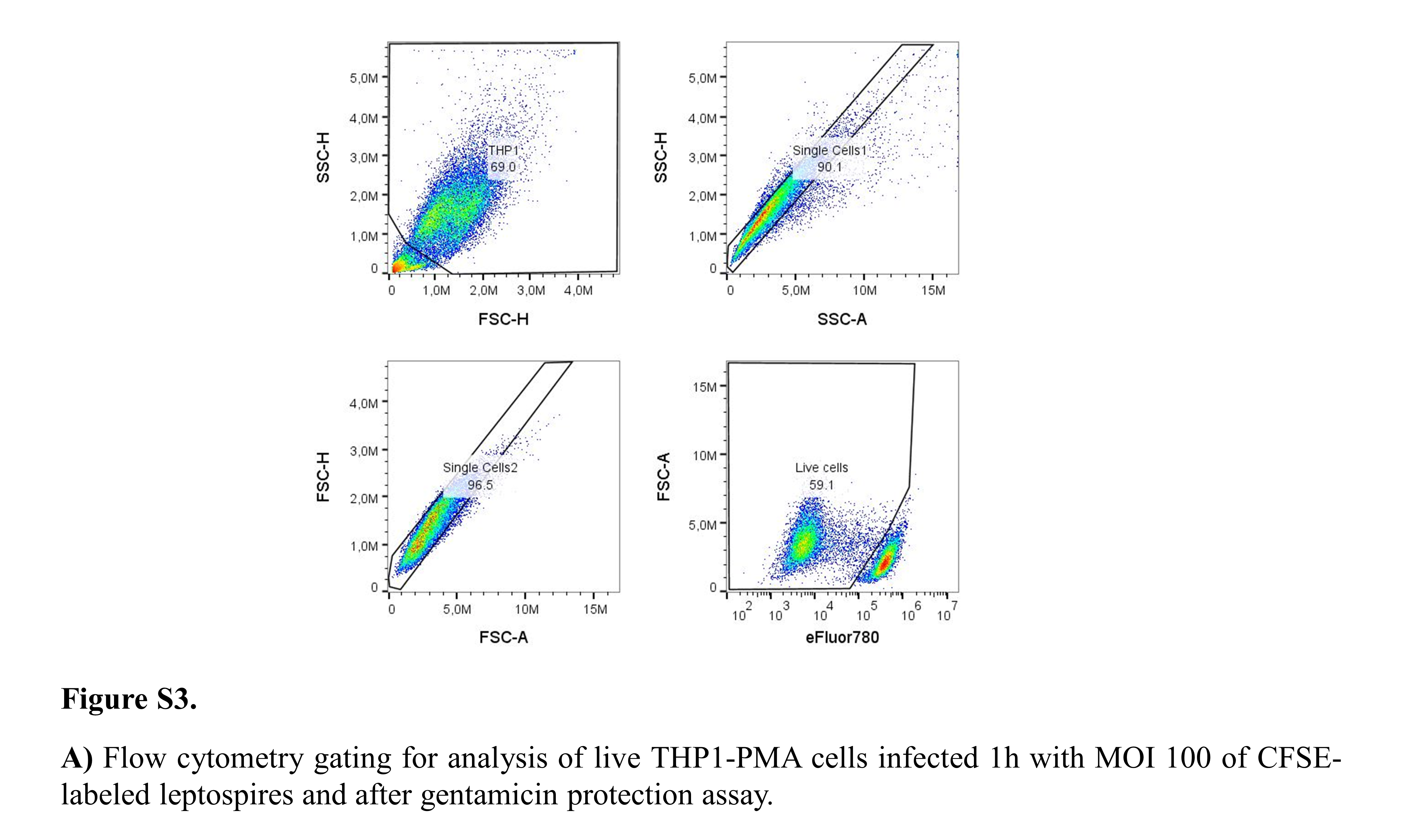

Supplement: Supplementary file 3 [file Image_3.tif]

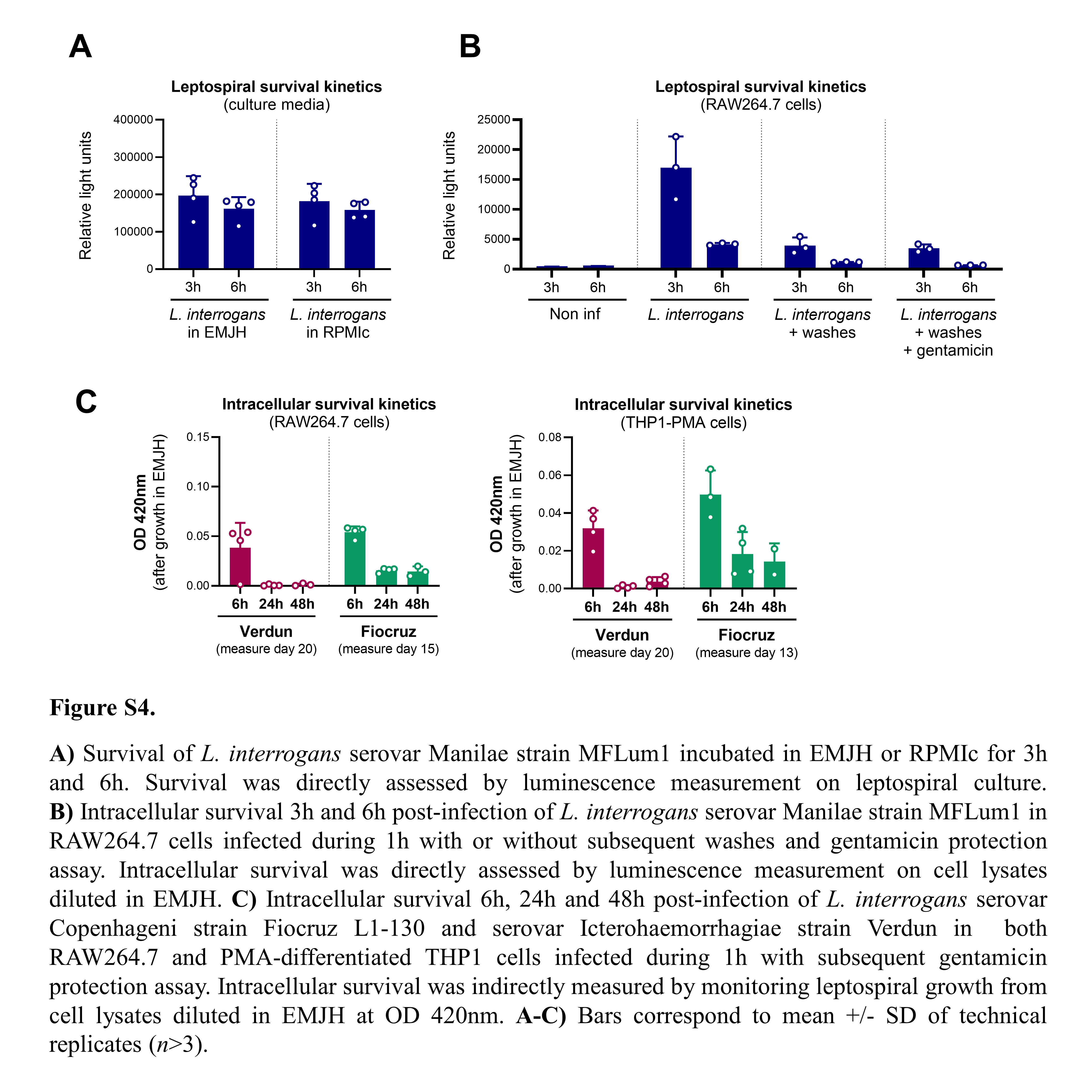

Supplement: Supplementary file 4 [file Image_4.tif]

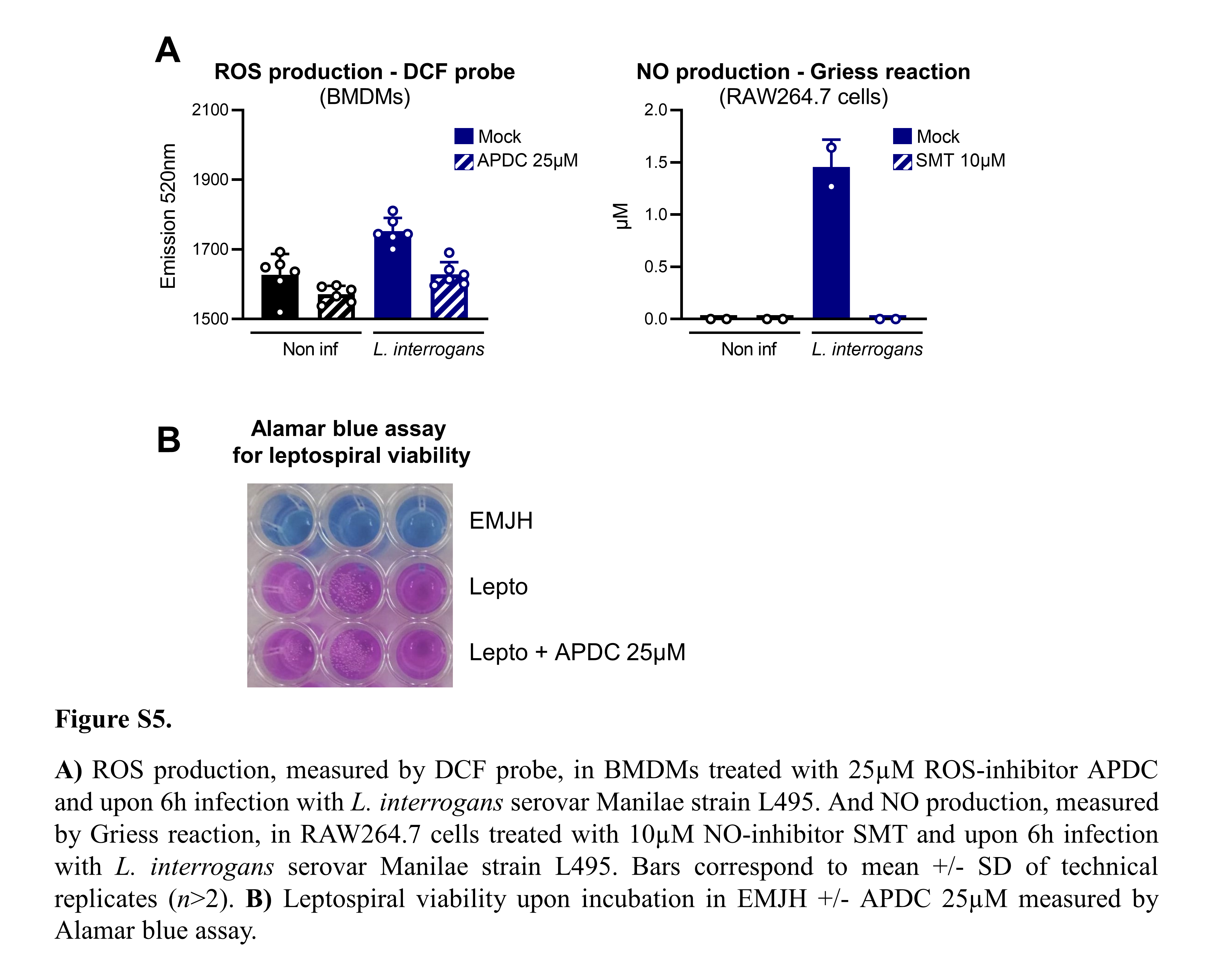

Supplement: Supplementary file 5 [file Image_5.tif]
